# Gastric Glomus Tumor: A Clinicopathologic and Immunohistochemical Study of 21 Cases

**DOI:** 10.1155/2020/5637893

**Published:** 2020-04-03

**Authors:** Jun Lin, Juan Shen, Hao Yue, Qiongqiong Li, Yuqing Cheng, Mengyun Zhou

**Affiliations:** ^1^Department of Pathology, Shanghai General Hospital, Shanghai Jiaotong University School of Medicine, No.100 Hai Ning Road, Hongkou District, Shanghai 200080, China; ^2^Department of Pathology, Changzhou Second People's Hospital, Nanjing Medical University, Changzhou 213003, China; ^3^Department of Pathology, Longhua Hospital, Shanghai University of Traditional Chinese Medicine, Shanghai 200032, China

## Abstract

Gastric glomus tumors (GGTs) are rare mesenchymal tumors. Most glomus tumors occur in the distal parts of the extremities. Here, we retrospectively analyzed the features of GGTs from two institutions. The histologic and clinical findings of all GGT cases from 2009 to 2018 were reviewed. The most common location was the antrum, the mean age of patients was 49.3 years, and the mean tumor size was 2.1 cm. Microscopically, small, round cell nodules surrounded the expansion of blood vessels in a nest pattern. Immunohistochemical assays for vimentin and smooth muscle actin (SMA) were positive, and assays for H-caldesmon and calponin were partially positive. GGT is rare and easily misdiagnosed before operation. However, immunohistochemistry is useful for the differential diagnosis. The majority of GGTs are benign, and local surgery achieving complete resection is the most effective treatment method.

## 1. Introduction

Glomus tumors are mesenchymal tumors composed of cells resembling the modified smooth muscle cells of the normal glomus body [1]. These tumors most commonly occur in the peripheral soft tissues, especially in the distal parts of the extremities [2]. Glomus tumors include 3 components: glomus cells, blood vessels, and smooth muscle. According to the relative proportions of these 3 components, the glomus tumor can be divided into three subtypes by light microscopy examination: (1) Solid glomus tumors: this type comprises approximately 75% of glomus tumors and is composed of nests of glomus cells surrounding capillary-sized vessels. (2) Glomangioma: this type comprises approximately 20% of glomus tumors and is characterized by cavernous hemangioma-like vascular structures surrounded by small clusters of glomus cells. (3) Glomangiomyoma: this type is the rarest, with an overall structure similar to that of a solid tumor or hemangioma, but with a transition between typical cells and spindle cells resembling mature smooth muscle. Glomus tumors may occur in deep-seated, visceral locations throughout the body, including the lung, pancreas, liver, and gastrointestinal and genitourinary tract [3]. Here, we report the clinicopathologic and immunohistochemical features of gastric glomus tumors (GGTs) seen in two institutions, with the aim of achieving a better understanding of this rare tumor and providing a reference for clinical treatment.

## 2. Methods

### 2.1. Patient and Case Selection

After obtaining approval of the institutional review boards from the two participating institutions, we selected specimens from patients diagnosed with GGT between January 2009 and December 2018. Data were extracted and collected from the patients' electronic medical records and pathology reports and included age, sex, location, size of the lesion, histopathologic features, and clinical follow-up (when available). All specimens were fixed in 4% buffered formalin and routinely processed. Two pathologists performed a repeat review of the routine hematoxylin and eosin (HE) slides to confirm the diagnosis. Follow-up was performed in an office setting or by telephone interview.

### 2.2. Immunohistochemistry

Each surgical specimen was specifically resectioned; 4-*μ*m-thick sections were obtained from 10% formalin-fixed and paraffin-embedded tissue blocks, followed by immunohistochemical staining using the following commercially available antibodies: vimentin (dilution 1 : 200), smooth muscle actin (SMA, dilution 1 : 800), muscle-specific actin (MAS, dilution 1 : 100), calponin (dilution 1 : 300), H-caldesmon (dilution 1 : 100), CD34 (dilution 1 : 200), panCK (AE1/AE3, dilution 1 : 50), CD117 (dilution 1 : 100), LCA (dilution 1 : 100), S-100 protein (dilution 1 : 300), NSE (dilution 1 : 100), Chromogranin A (CgA, dilution 1 : 200), and Synaptophysin (Syn, dilution 1 : 100). Appropriate positive control samples were used for all procedures. Antibody binding was detected using the universal immunoperoxidase polymer method (EnVision-kit; Dako, Carpinteria, CA, USA). A Dako-automated immunohistochemistry system (Dako, Carpinteria, CA, USA) was used according to the manufacturer's protocol. The immunohistochemistry results were independently interpreted by 2 experienced pathologists.

### 2.3. Reticulin Fiber Staining

Gomori Methenamine Silver was used (BASO, Zhuhai, China).

## 3. Results

### 3.1. Clinical Features

The clinical features of the 21 patients are summarized in Table 1. The patients included 11 females and 10 males. Tumors ranged in size between 0.8 cm and 3.5 cm, with a mean size of 2.1 cm (median, 2 cm). The age at initial examination ranged from 25 to 68 years (mean, 49.3 years). Three asymptomatic patients were found by physical examination. Furthermore, due to epigastric discomfort for more than one month, the 20^th^ patient underwent an endoscopic biopsy for gastric adenocarcinoma, followed by total gastrectomy, and the pathological examination revealed a GGT.

### 3.2. Pathologic Features

**Figure 1:**
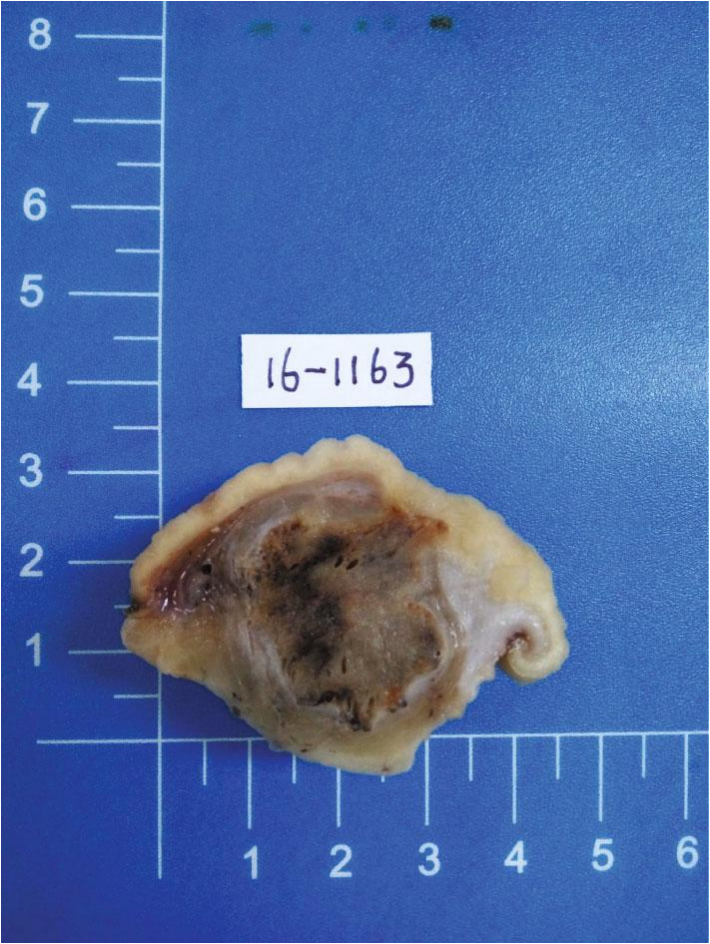
The tumor is localized in the submucosa and muscularis and has a clear boundary. The cross section of the tumor appears gray and grayish-red in color (case 19).

Macroscopically, the greatest diameter of the tumors was 0.8-3.5 cm. On the cut surface, the tumors were firm and solid or cystic and gray, grayish-red, grayish-white, or dark brown in color (Figure 1).

**Figure 2:**
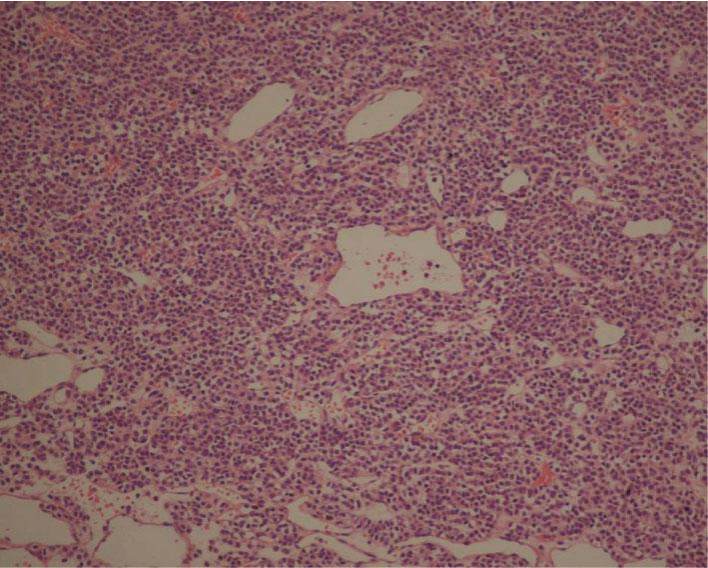
A solid arrangement of tumors around dilated blood vessels (HE ×100).

**Figure 3:**
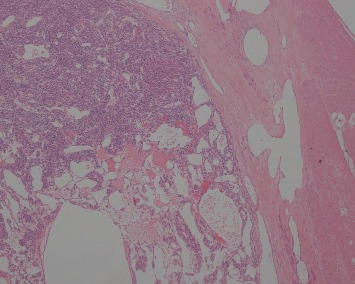
Cavernous hemangioma-like vascular structures in the tumor, which was well circumscribed. Dilated blood vessels in the muscularis around the mass (HE ×40).

**Figure 4:**
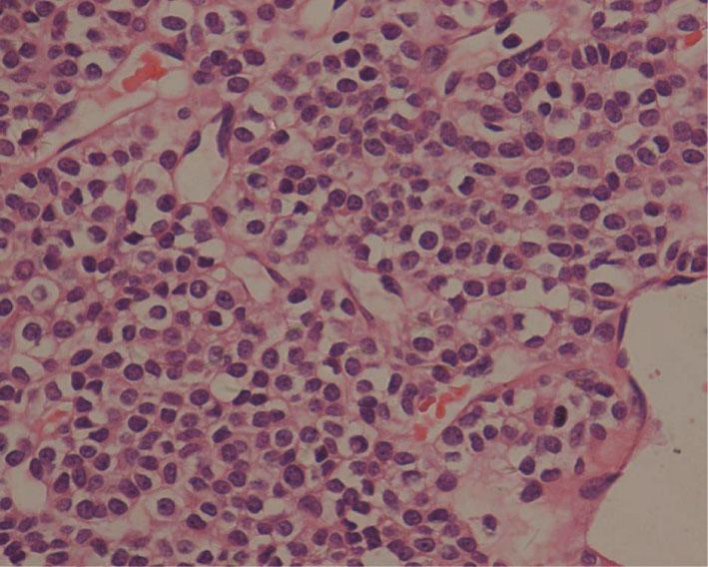
At high magnification, uniform, round, and clear tumor cells with sharp borders can be observed (HE ×400).

**Figure 5:**
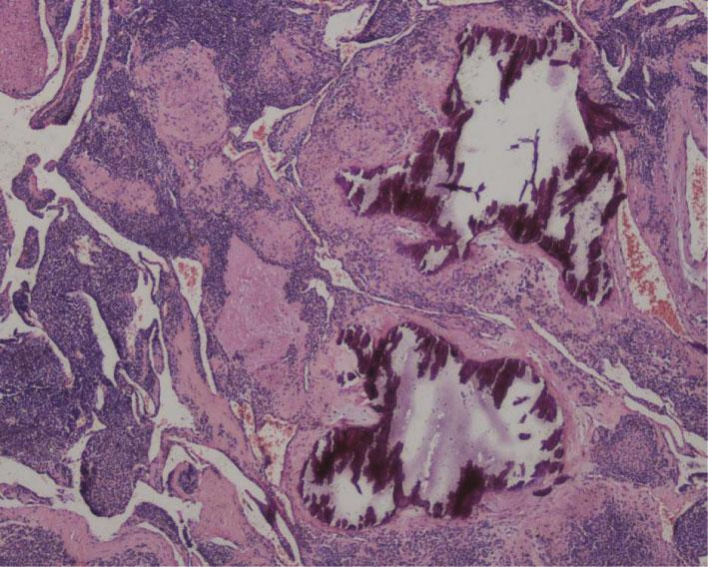
The stroma shows calcifications (HE ×40).

**Figure 6:**
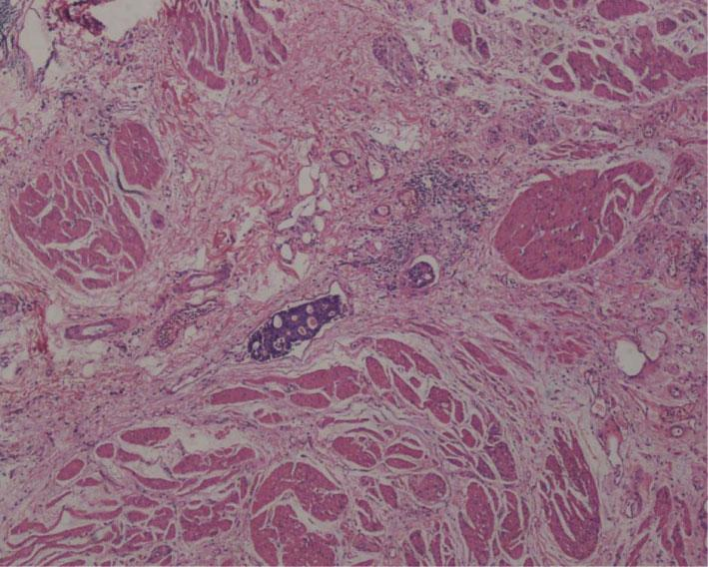
Cancer emboli are visible in the intramuscular vessels (case 20, HE ×40).

Histologically, the tumors were located in the gastric submucosa or muscularis and composed of glomus cells surrounding capillaries (Figure 2). Some tumors were well circumscribed (Figure 3), and the others had unclear boundaries. The glomus cells were small, uniform, and round without nuclear pleomorphism, mitotic figures, or necrosis (Figure 4). The stroma showed hyalinization or myxoid changes in some patients and ossification in one patient (Figure 5). Sporadic mast cells could be seen in the stroma. In many tumors, dilated blood vessels and/or lymphatic vessels were visible in the surrounding muscularis (Figure 3). In the 20^th^ patient, who had a gastric adenocarcinoma, intramuscular vascular dilatation could also be seen in the perigastric adenocarcinoma from a distance, and numerous cancer emboli were observed in the intramuscular vessels (Figure 6). Furthermore, this patient had 17 metastatic lymph nodes resulting from the adenocarcinoma.

**Figure 7:**
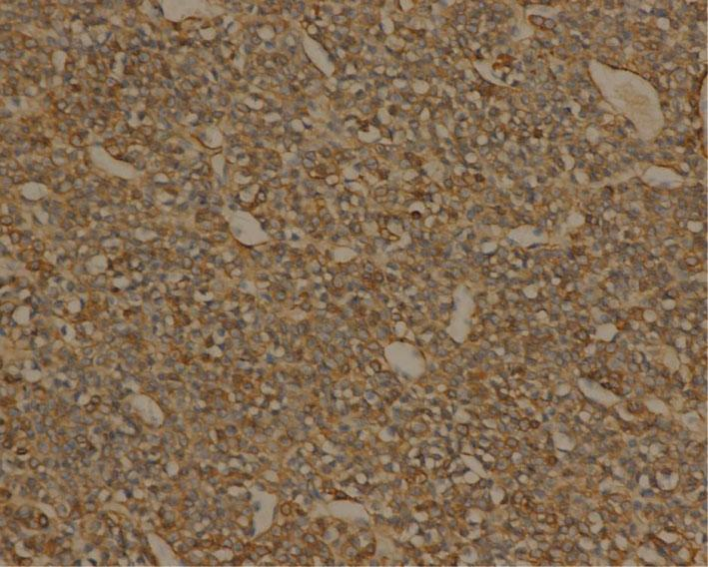
Tumor cells are positive for SMA upon immunohistochemical staining (EnVision ×200).

**Figure 8:**
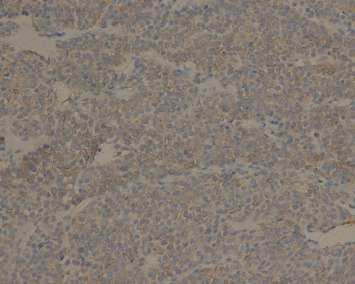
Syn is weakly expressed in the tumor cells (EnVision ×200).

Immunohistochemically, tumor cells in all 21 cases showed diffuse immunostaining for vimentin, SMA (Figure 7), MSA, and calponin. Partial expression of H-caldesmon was observed. Focal or more extensive positivity for Syn was observed in three cases (Figure 8), but all tumors were negative for CgA and NSE. The results for the remaining stains, including AE1/AE3, CD117, LCA, S100, and CD34, were all negative.

**Figure 9:**
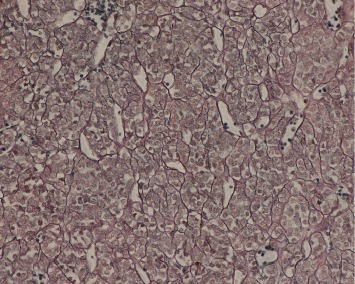
Reticulin fiber staining shows reticular fibers surrounding the tumor cells (reticulin fiber stains ×200).

Pericellular net-like positivity for reticulin fiber was nearly consistent (Figure 9).

### 3.3. Treatment and Follow-Up Data

As depicted in Table 1, the gastric tumors of 18 patients were excised by local resection (segmental resection or endoscopic resection). Subtotal gastrectomy was performed in two patients because of a suspected gastrointestinal stromal tumor. Total gastrectomy was performed due to gastric carcinoma in the 20^th^ patient.

Thirteen patients were followed up (13-118 months; median, 63.4 months), and no local recurrences were reported. However, the 20^th^ patient, who had gastric carcinoma, did not undergo chemotherapy or other adjuvant therapy and died 13 months after the operation.

## 4. Discussion

Glomus tumors are rare, accounting for fewer than 2% of soft tissue tumors. However, nearly 10% of cases involve multiple lesions [3]. This tumor is most common in the skin or superficial soft tissue, particularly in the subungual region, and can be found in deep soft tissue and internal organs (such as the nerves, bone, penis, bladder, mediastinum, gastrointestinal tract, liver, and cervix) [4, 5]. Glomus tumors that occur in the stomach are relatively rare, and the onset age is 19-90 years. Most tumors occur in middle-aged and elderly people, mostly in women, and the most common site is the antrum. In our cohort, the numbers of males and females were nearly equal. Patients are often treated for symptoms such as epigastric discomfort, epigastric pain, and upper gastrointestinal hemorrhage [6]. Malignant glomus tumors are quite rare, accounting for fewer than 1% of glomus tumors, and they generally have a deep location but can also be located on the skin [7].

Most GGTs are located in the submucosa and muscularis propria of the stomach, and mucosal elevation is usually visible under a gastroscope. The tumor does not generally involve the mucosa, but some cases involve erosion of the mucosal surface, and most tumors are diagnosed as gastrointestinal stromal tumors before surgery. The diagnosis depends on pathological morphology and immunohistochemistry results.

The vast majority of glomus cells does not exhibit atypia; are small, round, and uniform in size; have clear boundaries and clear cytoplasm; and are pale or slightly eosinophilic with round and centered nuclei, fine chromatin, and unclear nucleoli. Occasionally, neoplastic cells will exhibit atypia or venous invasion but no other adverse manifestations, such as mitotic activity and/or pathologic mitosis, and these cells are considered benign. Hyaline or mucous degeneration often occurs in the tumor stroma, with occasional calcification or ossification. In addition, reticular fiber staining shows reticular fiber around tumor cells, cell nests, and vessels. Both electron microscopy and immunohistochemical observation show characteristics of smooth muscle differentiation. Under electron microscopy, the cytoplasm is found to contain myofilamentous dense bodies, with connecting structures between adjacent cells and the basement membrane around the cells [8]. Immunohistochemical staining shows that tumor cells are positive for SMA, vimentin, H-caldesmon, and calponin. Syn and CD34 can be positive [4, 9, 10].

Most gastric neoplasms can be diagnosed according to these histological manifestations. In our study, thirteen cases were solid glomus tumors (Figure 2), and three cases were glomangiomas. Another five cases were both (Figure 3). No cases involved glomangiomyoma. Both vimentin and SMA were diffusely positive, and both H-caldesmon and calponin were partially positive. Three cases were positive for Syn.

Differential diagnosis: [1] Gastrointestinal stromal tumor: this type of tumor exhibits endoscopic findings similar to those of GGT; the cells under the stroma are fusiform and short fusiform, with epithelioid manifestations. The stroma is not rich in blood vessels or dilated veins. Immunohistochemistry results for CD117, dog-1, and CD34 are positive, and SMA is positive but not as strongly as in glomus tumors. [2] Neuroendocrine tumor, G1 (carcinoid): the microscopic tumor tissue is rich in the blood sinus. Tumor cells are consistent in size and are arranged in a nest-like pattern but are carcinoid in the gastric mucosa or submucosa. Tumor cells have limited cytoplasm, nuclear chromatin is relatively coarse, and cell borders are not clear. The cells show atypia. Immunohistochemical assays for CgA, Syn, and panCK are positive, and SMA and vimentin negativity can be identified [11]. [3] Paraganglioma: paraganglioma in the stomach is rare. The tumor cells are composed of chief cells and sustentacular cells, which are arranged in nests and found in organs around the blood vessels. Immunohistochemistry assays of the chief cells are positive for CgA and Syn, and the sustentacular cells are positive for S-100. (4) Hemangiopericytoma: dilated blood vessels can be seen in both hemangiopericytoma and GGT, but the blood vessels of hemangiopericytoma are mostly antler-like. The surrounding cells are fusiform, CD34 is positive, and smooth muscle markers are negative [4, 12]. (5) Lymphoma: it is difficult to distinguish lymphoma from GGT in frozen sections. The tumor cell size is relatively consistent, and the cells are diffusely arranged. Lymphoma can be easily identified by immunohistochemistry.

Treatment and prognosis: glomus tumors are benign tumors that can be simply excised and have a recurrence rate of 10% [13]. Atypical or malignant glomus tumors are exceedingly rare and occur more frequently as deep-seated, large tumors in the gastrointestinal system [4]. Folpe et al. [14] proposed the following diagnostic criteria of malignant glomus tumors: >2 cm in diameter with a deep location, visible pathological mitosis, nuclear atypia, and >5 mitotic cells/50 HPF. Of the above malignant indicators, if the tumor has only >5 mitotic cells/50 HPF and the location is superficial, or if it only has a large size or only a deep location, it can be classified as a glomus tumor with uncertain malignant potential.

Some studies suggest that the above criteria are not suitable for GGTs. GGTs are deep soft tissue tumors, and the maximum tumor diameter in more than half of patients is greater than 2 cm [4, 15, 16]. In addition, a total of 11 patients with malignant GGTs have been reported in the literature with tumor sizes ranging from 3 to 14 cm, and 8 of the tumors were larger than 5 cm [14, 4, 17, 7, 18, 19, 20, 21, 22, 23]. Therefore, a size of 5 cm might be a more appropriate indicator of risk for GGTs [4].

Surgical resection of most GGTs has a good prognosis. In our study, the maximum diameter of 10 tumors was greater than 2 cm, and the tumor boundary was unclear. After local resection, five patients were followed up for 13-98 months, and their last reported condition was good.

The comprehensive literature reports that only a few cases involved metastases, and the vast majority of cases presented benign processes [4, 16]. We believe that patients with large tumors, high heteromorphism, and active mitosis should undergo long-term follow-up to accumulate additional data for the diagnosis of malignant GGTs and to reduce unnecessary surgeries. To the best of our knowledge, this is the first report of a case of a GGT with gastric adenocarcinoma in the English language literature. The patient died thirteen months after surgery. An earlier paper reported a case of GGT with lymphoma [24]. The patient developed bone marrow involvement shortly after surgery. These two cases indicate that the prognosis of patients with a GGT accompanied by other malignant tumors is poor and may be related to the dilatation of gastric wall blood vessels caused by the GGT.

## 5. Conclusion

In summary, we analyzed the clinicopathologic and immunohistochemical features of 21 GGTs. Most GGTs are clinically benign, and immunohistochemistry is useful for the differential diagnosis. A local operation achieving complete resection is the most effective treatment method. However, follow-up data were not available in eight of our cases (approximately 38%), including the tumor with the largest size (3.5 cm). Therefore, it is necessary to accumulate additional cases and follow-up data. However, our data show that patients with gastric adenocarcinoma might have a poor prognosis. Therefore, given the rarity of this tumor, identifying more cases will help us better understand its prognosis.

## Figures and Tables

**Table 1:** Clinicopathologic features of 21 patients with gastric glomus tumors.

No	Gender	Age	Symptom	Gastric location	Site	Size (cm)	Follow-up (months)	Operation methods
1	F	59	Epigastric discomfort	Antrum	Mucosa to muscularis	3.5		Segmental resection
2	F	51	Epigastric discomfort	Antrum	Submucosa to muscularis	1.5		Segmental resection
3	F	44	Epigastric discomfort	Antrum	Submucosa to muscularis	2.5		Segmental resection
4	M	62	Epigastric pain	Antrum	Submucosa to muscularis	1.5		Segmental resection
5	M	40	None	Antrum	Submucosa to muscularis	2.3		Segmental resection
6	F	25	Melena	Antrum	Submucosa to muscularis	2.6		Segmental resection
7	M	46	None	Antrum	Submucosa to muscularis	2.5		Segmental resection
8	M	43	Epigastric discomfort	Antrum	Submucosa to muscularis	2.0		Segmental resection
9	M	54	Epigastric discomfort	Antrum	Submucosa to muscularis	2.3	73	Subtotal gastrectomy
10	F	40	Epigastric discomfort	Antrum	Submucosa to muscularis	2.0	60	Segmental resection
11	F	34	Epigastric pain	Antrum	Muscularis	2.0	97	Endoscopic resection
12	F	37	Epigastric discomfort	Body	Mucosa to submucosa	1.5	84	Subtotal gastrectomy
13	M	54	Epigastric pain	Antrum	Submucosa to muscularis	0.8	58	Endoscopic resection
14	M	60	Epigastric pain	Antrum	Mucosa to muscularis	2.7	48	Endoscopic resection
15	M	45	Epigastric discomfort	Antrum	Submucosa to muscularis	1.2	118	Segmental resection
16	M	60	Epigastric discomfort	Antrum	Muscularis	2.3	98	Segmental resection
17	F	42	Epigastric pain	Antrum	Submucosa to muscularis	1.5	66	Segmental resection
18	M	55	Epigastric discomfort	Antrum	Submucosa to muscularis	1.5	59	Segmental resection
19	F	61	Melena	Antrum	Submucosa to muscularis	2.8	37	Segmental resection
20	F	68	Epigastric discomfort	Antrum	Submucosa to muscularis	1.5	13	Total gastrectomy
21	F	55	None	Antrum	Submucosa to muscularis	2.7	13	Endoscopic resection

## Data Availability

The data is available through contact by the corresponding author Dr. Mengyun Zhou if there are no commercial interests.

## References

[1] Folpe A. L., Brema H., Legius E. (2013). Glomus tumours. *World Health Organization classification of tumours of soft tissue and bone*.

[2] Tsuneyoshi M., Enjoji M. (1982). Glomus tumor: a clinicopathologic and electron microscopic study. *Cancer*.

[3] Yoo Y. S., Choi J. H., Heo G., Kim S. W., Kim H.-J. (2011). Double glomus tumors originating in the submandibular and parotid regions. *Clinical and Experimental Otorhinolaryngology*.

[4] Miettinen M., Paal E., Lasota J., Sobin L. H. (2002). Gastrointestinal glomus tumors: a clinicopathologic, immunohistochemical, and molecular genetic study of 32 cases. *The American Journal of Surgical Pathology*.

[5] Lee H. W., Lee J. J., Yang D. H., Lee B. H. (2006). A clinicopathologic study of glomus tumor of the stomach. *Journal of Clinical Gastroenterology*.

[6] Fang H. Q., Yang J., Zhang F. F., Cui Y., Han A. J. (2010). Clinicopathological features of gastric glomus tumor. *World Journal of Gastroenterology*.

[7] Bray A. P. J. J., Wong N. A. C. S., Narayan S. (2009). Cutaneous metastasis from gastric glomus tumour. *Clinical and Experimental Dermatology*.

[8] Souza F. F., Chen E. (2009). Mesenchymal cystic hamartoma of the lung: MRI and PET/CT appearance. *Journal of Thoracic Imaging*.

[9] Matevossian E., Brücher B. L. D. M., Nährig J., Feubner H., Hüser N. (2008). Glomus tumor of the stomach simulating a gastrointestinal stromal tumor: a case report and review of literature. *Case Reports in Gastroenterology*.

[10] Xu X. D., Lu X. H., Ye G. X., Hu X. R. (2010). Immunohistochemical analysis and biological behaviour of gastric glomus tumours: a case report and review of the literature. *The Journal of International Medical Research*.

[11] Vig T., Bindra M. S., Kumar R. M., Alexander S. (2017). Gastric glomus tumour misdiagnosed as gastric carcinoid: an unfamiliar entity with aids to diagnosis and review of literature. *Journal of Clinical and Diagnostic Research*.

[12] Kapur U., Hobbs C. M., McDermott E., Mooney E. E. (2004). Gastric glomus tumor. *Annals of Diagnostic Pathology*.

[13] Mravic M., LaChaud G., Nguyen A., Scott M. A., Dry S. M., James A. W. (2015). Clinical and histopathological diagnosis of glomus Tumor. *International Journal of Surgical Pathology*.

[14] Folpe A. L., Fanburg-Smith J. C., Miettinen M., Weiss S. W. (2001). Atypical and malignant glomus tumors: analysis of 52 cases, with a proposal for the reclassification of glomus tumors. *The American Journal of Surgical Pathology*.

[15] Warner K. E., Haidak G. L. (1984). Massive glomus tumor of the stomach: 20-year follow-up and autopsy findings. *The American Journal of Gastroenterology*.

[16] Haque S., Modlin I. M., West A. B., Path M. R. (1992). Multiple glomus tumors of the stomach with intravascular spread. *The American Journal of Surgical Pathology*.

[17] Lee H., Choi Y. S., Oh S. C. (2009). Malignant glomus tumors of the stomach - a report of 2 cases with multiple metastases. *The Korean Journal of Pathology*.

[18] Song S. E., Lee C. H., Kim K. A., Lee H. J., Park C. M. (2010). Malignant glomus tumor of the stomach with multiorgan metastases: report of a case. *Surgery Today*.

[19] Teng L., Ke C., Yan M., Hou W., Tian D. (2012). Atypical glomus tumor of the body of stomach: a case report and review of literature. *The Chinese-German Journal of Clinical Oncology*.

[20] Teng T. H., Huang S. H., Liang C. W. (2012). Malignant gastric glomus tumour mimicking GIST. *Pathology*.

[21] Abu-Zaid A., Azzam A., Amin T., Mohammed S. (2013). Malignant glomus tumor (glomangiosarcoma) of intestinal ileum: a rare case report. *Case Reports in Pathology*.

[22] Zaidi S., Arafah M. (2016). Malignant gastric glomus tumor: a case report and literature review of a rare entity. *Oman Medical Journal*.

[23] Toti L., Manzia T. M., Roma S. (2019). Rare malignant glomus tumor of the stomach with liver metastases. *Radiology Case Reports*.

[24] West A. B., Buckley P. J. (1992). Mantle zone lymphoma in a gastric glomus tumor. *Cancer*.

